# IKKα and IKKβ Each Function to Regulate NF-κB Activation in the TNF-Induced/Canonical Pathway

**DOI:** 10.1371/journal.pone.0009428

**Published:** 2010-02-25

**Authors:** Mazhar Adli, Evan Merkhofer, Patricia Cogswell, Albert S. Baldwin

**Affiliations:** 1 Department of Biology, University of North Carolina, Chapel Hill, North Carolina, United States of America; 2 Curriculum in Genetics and Molecular Biology, University of North Carolina, Chapel Hill, North Carolina, United States of America; 3 Lineberger Comprehensive Cancer Center, University of North Carolina, Chapel Hill, North Carolina, United States of America; Cinvestav, Mexico

## Abstract

**Background:**

Activation of the transcription factor NF-κB by cytokines is rapid, mediated through the activation of the IKK complex with subsequent phosphorylation and degradation of the inhibitory IκB proteins. The IKK complex is comprised of two catalytic subunits, IKKα and IKKβ, and a regulatory protein known as NEMO. Using cells from mice that are genetically deficient in IKKβ or IKKα, or using a kinase inactive mutant of IKKβ, it has been proposed that IKKβ is critical for TNF-induced IκB phosphorylation/degradation through the canonical pathway while IKKα has been shown to be involved in the non-canonical pathway for NF-κB activation. These conclusions have led to a focus on development of IKKβ inhibitors for potential use in inflammatory disorders and cancer.

**Methodology:**

Analysis of NF-κB activation in response to TNF in MEFs reveals that IKKβ is essential for efficient phosphorylation and subsequent degradation of IκBα, yet IKKα contributes to the NF-κB activation response in these cells as measured via DNA binding assays. In HeLa cells, both IKKα and IKKβ contribute to IκBα phosphorylation and NF-κB activation. A kinase inactive mutant of IKKβ, which has been used as evidence for the critical importance of IKKβ in TNF-induced signaling, blocks activation of NF-κB induced by IKKα, even in cells that are deficient in IKKβ.

**Conclusions:**

These results demonstrate the importance of IKKα in canonical NF-κB activation, downstream of cytokine treatment of cells. The experiments suggest that IKKα will be a therapeutic target in inflammatory disorders.

## Introduction

The transcription factor nuclear factor-kappaB (NF-κB) plays critical roles in inflammation, control of cell death pathways and cell proliferation which are hallmarks of many human diseases [1]–[3]. The mammalian NF-κB transcription factor is a family of 5 proteins comprised of NF-κB1 (p50/p105), NF-κB2 (p52/p100), c-Rel, RelB, and RelA (p65). These proteins exist as homo- or heterodimers bound by inhibitory κB (IκB) proteins under unstimulated conditions [3]. In unstimulated cells, NF-κB is tightly regulated by one of several inhibitors of NF-κB (IκBα, β, ε) [1]–[4]. A large number of intra- and extra-cellular stimuli, including cytokines, PMA, bacterial LPS, viral infection, stress-induced responses, and T and B cell activation, lead to NF-κB activation. NF-κB activation involves IκB kinase (IKK) activation which leads to IκB phosphorylation and subsequent ubiquitin-dependent IκB degradation by the 26S proteosome complex [1]–[4]. The released NF-κB transcription factor with unmasked nuclear localization signal then accumulates in the nucleus to regulate the expression of genes encoding cytokines, cytokine receptors, and apoptotic regulators [1]–[4].

IκB phosphorylation by the high molecular weight IκB kinase (IKK) complex (approximately 700 kDa) is a critical regulatory step in the NF-κB activation pathway [1]–[5]. This kinase complex was partially identified initially in unstimulated Hela cells and was later found to be activated in cells treated with TNFα [6]. Subsequently several groups identified two highly related kinases named IKK1/IKKα and IKK2/IKKβ as the catalytic components of this complex [6]–[8]. Both of these kinases have been shown to have specificity for serines 32 and 36 in the N-terminus of IκBα with phosphorylation leading to ubiquitination and degradation of this inhibitory protein [9]. In addition to IKKα and IKKβ, a non-catalytic, regulatory component of IKK was also identified and called NF-κB Essential modifier (NEMO) or IKKγ [10], [11]. Additionally, it has been reported that both IKKα and IKKβ can phosphorylate the RelA/p65 subunit to promote transactivation potential [12].

Insight into the physiological roles of the two catalytic IKK subunits comes from gene targeting studies. IKKβ knockout mice display a phenotype similar or identical to knockout of RelA, namely embryonic lethal with severe liver apoptosis [13]–[15]. A similar phenotype was seen in the NEMO/IKKγ knockout animal [16]. Mouse embryonic fibroblast cells that were isolated from IKKβ deficient embryos showed a marked reduction in TNFα- and interleukin-1alpha-induced NF-κB activity, as measured by EMSA and by effects on IκB degradation. The IKKβ −/− knockout cells exhibit significantly enhanced apoptosis in response to TNFα [13]–[15]. Importantly, IKK activity directed to phosphorylation of IκB in vitro was essentially lost in IKKβ null cells [13]–[15]. A role of IKKα in classical NF-κB signaling is less clear compared to IKKβ. IKKα deficient mice exhibit abnormal morphogenesis and developmental defects [17]–[19]. Consistent with conclusions derived using IKKβ −/− fibroblasts, IKKα does not seem to have a significant influence on cytokine-induced IKK activity directed to IκBα [17], [18]. However, IKKα-deficient mouse embryonic fibroblast (MEF) cells exhibited reduced NF-κB activation as measured by EMSA in response to cytokine treatment [17], [18]. Another group did not find reduced cytokine-induced NF-κB DNA binding activity in IKKα −/− MEFs [19]. In the light of these genetic studies and additional biochemical studies, it has been generally assumed that IKKβ but not IKKα is the primary regulator of NF-κB dependent proinflammatory signal transduction [1]–[5]. On the other hand, IKKα is known to be essential in non-canonical NF-κB activation by regulating p100 precursor processing and activation of the p52/RelB heterodimer [1]–[5]. Recently, we and others have demonstrated that IKKα has an important nuclear function by regulating the control of target genes at the level of histone phosphorylation [20], [21]. Interestingly, the observation that hepatocyte-specific ablation of IKKβ did not lead to impaired activation of NF-κB by TNF as measured by gel shift assay and IκB degradation [22] suggests the involvement of another kinase in the canonical pathway at least in adult hepatocytes. Here we have explored individual roles of IKKα and IKKβ in canonical NF-κB activation in MEF cells as well as cancer cells. Our results suggest that IKKα, like IKKβ, is critical for efficient cytokine-induced NF-κB activation. In fibroblasts IKKα is not significantly involved in IκBα phosphorylation/degradation, yet contributes to activation of NF-κB through an unknown mechanism in these cells. In HeLa cells, IKKα and IKKβ each contribute to IKK activity directed to IκBα to control its phosphorylation and subsequent degradation. Expression of a kinase inactive variant of IKKβ, which has been used previously to provide evidence for the importance of IKKβ in the canonical pathway, is shown here to block IKKα activity. These studies suggest that inhibition of IKKα is a rational approach in blocking inflammatory disorders.

## Materials and Methods

### Reagents and Materials

Mouse embryonic fibroblast (MEF) and HeLa cells were cultured in Dulbecco's modified Eagle's medium (DMEM), complemented with 10% fetal calf serum (FCS), 100 units/ml penicillin, 100 µg/ml streptomycin. SKBr3 cells were cultured in McCoy's 5A medium complemented with 10% fetal calf serum (FCS), 100 units/ml penicillin and 100 µg/ml streptomycin. Wild type, IKKα, IKKβ single and IKKα/β double knockout cells (DKO) were the kind gift from Dr. Inder Verma. Antibodies to phospho-specific NF-κB p65 (Ser-536) and IκBα (Ser 32/36) were obtained from Cell Signaling. Antibodies to β-tubulin and to IκBα were obtained from Santa Cruz. Antibodies to IKKα and IKKβ were obtained from Upstate Biotechnology Inc. RhTNF-α (Promega) was used at a final concentration of 10 ng/ml.

### Western Blot

After stimulation, cultured cells were lysed on ice for 5 min in RIPA lysis buffer with freshly added protease and phosphatase inhibitor cocktails. Lysates were cleared by centrifugation at 4 °C for 15 min at 13,000 *g*. The amount of total protein was measured and equal amounts (20 µg) were fractionated by NuPAGE Novex 4–12% Bis Tris gels (Invitrogen) and electro-transferred to polyvinylidene difluoride membranes. Membranes were blotted with the indicated antibodies, and proteins were detected using an enhanced chemiluminescence detection system (Amersham Biosciences, Freiburg, Germany). Where indicated, membranes were stripped and re-probed with the indicated antibody.

### Electrophoretic Mobility Shift Assay (EMSA)

EMSAs were performed as previously described [23]. Briefly, 4−5 µg of nuclear extracts, prepared following cell stimulation, were incubated with a radiolabeled DNA probe containing an NF-κB consensus site. For supershifts, 1 µl of anti-p65 antibody (Rockland) or 2 µl of anti-p50 antibody (Santa Cruz, SC-7178) was added and the binding reaction was allowed to proceed for an additional 15 min. Protein−DNA complexes were resolved on a non-denaturing polyacrylamide gel and visualized by autoradiography.

### siRNA Knockdown Experiments

IKKα and IKKβ mRNA were knocked down with siRNA obtained from Dharmacon. Dharmafect 1 (Dharmacon Company) transfection reagent was used for all si-RNA transfection as described in the manufacturer's protocol. SiRNA was transfected for 72 hrs, and lysate preparation and westerns were performed as described [24].

### Luciferase Assays

SKBr3 cells stably expressing the 3x-κB plasmid were plated in equal number in triplicate in 24-well plates and transfected with siRNA for 72 hours. Cells were lysed in MPER and luciferase activity was measured with Promega Luciferase Assay System (Promega). Luciferase levels were normalized by protein concentration using a Bradford assay. MEF cells were seeded in 24-well plates at 30–50% density and transfected the next day with the indicated expression vectors and 3x-κB Luciferase reporter gene for 48 h using Effectene (Qiagen) transfection reagent according to the manufacturer's instruction. β-galactosidase reporter gene was used as an internal control. The total amount of transfected DNA (500 ng of DNA) in each well was adjusted by adding empty plasmid vector (pcDNA3.1). Where indicated, 100 ng and 200 ng of IKKβ KM vector has been used. Luciferase activity of whole cell lysates was measured by using a luciferase assay kit (Promega). β-galactosidase activity was measured by liquid -galactosidase assay with chlorophenolred-β-D-galactopyranoside substrate. Relative luciferase activity was calculated by normalizing the assay results to β-galactosidase expression values. Luciferase-fold induction was calculated by normalizing the results to control treatment, which was assumed as 1-fold induction. HeLa cells, seeded in 24-well plates were transiently transfected with the indicated siRNAs for 48 hr. Media was then replaced and cells were further transfected with the NF-κB response 3X-κB luciferase reporter and a control Renilla luciferase construct. 24 hr later, cells were lysed and dual luciferase assays were performed. Luciferase readings in untreated and control vector transfected cells were normalized to 1.

## Results

### TNF-Induced NF-κB Activity Is Diminished in IKKα as Well as IKKβ Deficient MEF Cells

**Figure 1:**
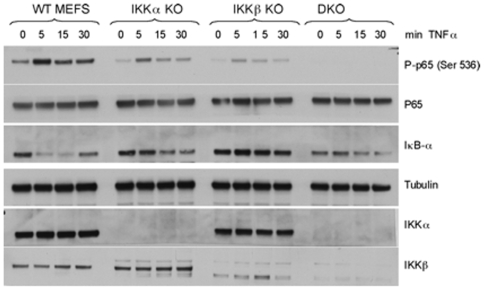
The role of IKKα and IKKβ in p65 phosphorylation and IκBα degradation in response to TNFα. MEF cells that are deficient for IKKα, IKKβ, or both IKKα and IKKβ (DKO) were treated with TNFα for the indicated times. NF-κB activity, as measured by IκBα degradation and p65 phosphorylation, is diminished in IKKα and IKKβ deficient MEF cells. IKKα and IKKβ DKO cells show no detectable p65 phosphorylation. Tubulin levels are shown as a loading control.

Experiments were initiated to assay roles of IKKα and IKKβ in inducing IκBα phosphorylation and activation of NF-κB in response to a well-studied NF-κB inducer, TNFα. For this purpose, mouse embryonic fibroblast cells (MEFs) that are deficient for IKKα or for IKKβ singly as well as IKKα/β double knock-out cells have been utilized. As shown in Figure 1, TNFα induces expected p65 phosphorylation at Ser 536 position as well as IκBα degradation in as early as 5 minutes post-stimulation. Importantly, there is diminished p65 phosphorylation in both IKKα and IKKβ deficient MEF cells. Interestingly, lack of IKKα delayed IκBα degradation while lack of IKKβ significantly suppressed the TNF-induced degradation of IκBα. Relative to the IKKβ deficient cells, IκBα appears weakly degraded at the 30 minute time point (Fig. 1) but by 60 minutes these levels return (data not shown, and also see ref. 15). IKKα/β DKO MEFs have near complete loss of p65 phosphorylation and IκBα degradation, as expected (note lower levels of IκBα in these cells, indicating significantly reduced NF-κB-dependent transcription of its inhibitor). These results (Figure 1) demonstrate that both IKKα and IKKβ are required for efficient NF-κB activation in MEFs as measured by p65 phosphorylation at Ser536, yet IKKβ appears to be significantly more important in the IκBα phosphorylation/degradation response in fibroblasts. This work is consistent with previous work [13]–[15], [17]–[19] which showed that IKK *in vitro* activity, directed to recombinant IκBα, is not diminished in IKK1 (IKKα) null MEFs, yet is significantly reduced in IKK2/IKKβ null cells.

### NF-κB DNA Binding Activity Is Diminished in both IKKα and IKKβ Deficient MEF Cells

**Figure 2:**
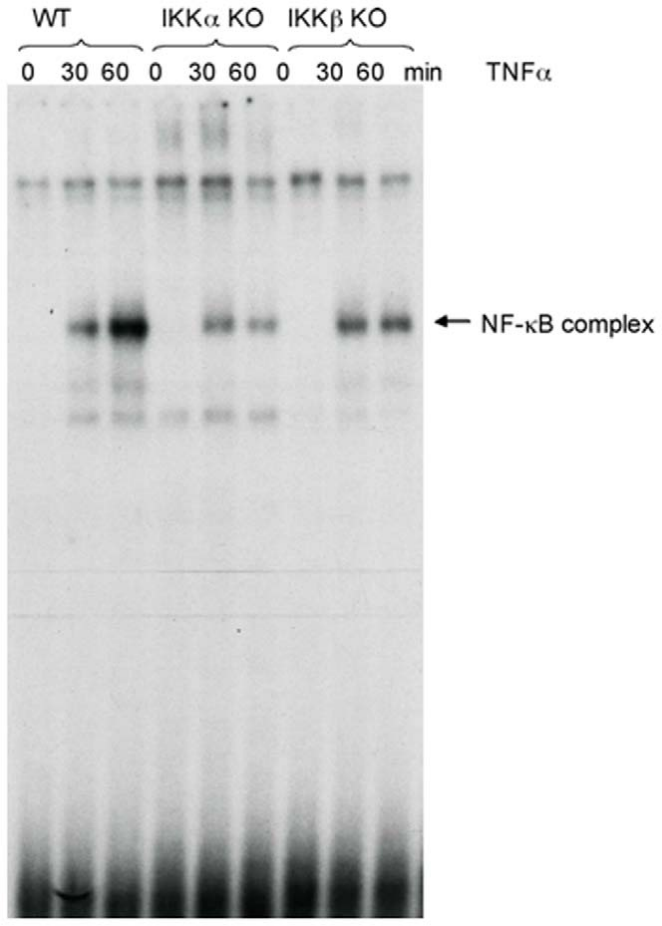
NF-κB DNA binding activity is reduced in IKKα and in IKKβ deficient cells. DNA binding activity of NF-κB was measured by gel shift assay. Indicated MEF cells were treated with TNFα for indicated times. Nuclear proteins were subject to gel shift assay for DNA binding analysis.

In addition to IκBα degradation and p65 phosphorylation, we have studied whether IKKα and IKKβ differentially affect NF-κB DNA binding activity in response to TNF as measured by EMSA/gel shift assay. NF-κB DNA binding activity was investigated in WT, IKKα −/−, and IKKβ −/− cells. As shown in Figure 2, there is significant induction of NF-κB (p50/p65) DNA binding activity in response to TNFα in WT MEF cells. However, this DNA binding activity is diminished in both IKKα and IKKβ deficient cells. The level and the kinetics of NF-κB DNA binding activity is comparable in IKKα and IKKβ deficient MEFs cells. This data suggests that IKKα, as well IKKβ, is essential for optimal NF-κB DNA binding activity, potentially through different mechanisms (see Discussion). Promoter studies (see below) confirm a functional role for IKKα in TNF-induced NF-κB activation in MEF cells.

### Similar Roles for IKKα and IKKβ in Response to TNFα Induced NF-κB Activation in Hela Cells

**Figure 3:**
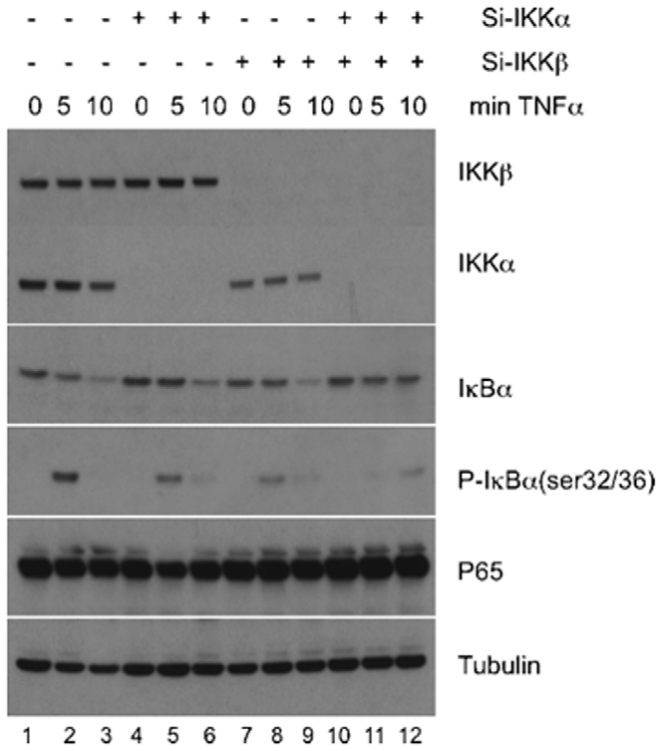
IKKα and IKKβ knock-down in Hela cells leads to diminished IκB degradation and p65 phosphorylation. HeLa cells were grown in 6-well plates and transfected with the indicated siRNA for 3 days. Western blots were performed on total cell extracts after treatment with TNF for the indicated times. Tubulin levels are shown as a loading control.

**Figure 4:**
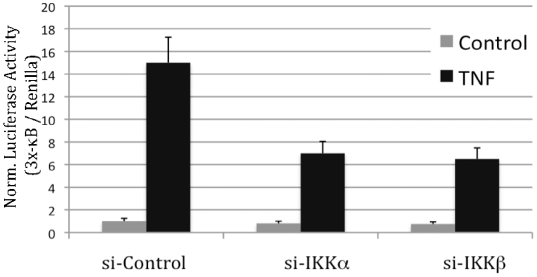
IKKα and IKKβ each contribute to TNF-induced NF-κB activity in HeLa cells. HeLa cells, seeded in 24-well plates were transiently transfected with indicated siRNA constructs for 48 hr. Then media was replaced and cells were further transfected with NF-κB responsive 3x-κB luciferase and a control Renilla luciferase contructs. TNF was added (as indicated) and 24 hr later cells were lysed and dual lucifearse assay was performed. Luciferase readings in untreated and control vector transfected cells were normalized as 1.

Most studies regarding the roles of IKKα and IKKβ have been performed in MEFs null for either subunit. To expand these studies, we have analyzed the differential roles of IKKα and IKKβ in response to TNF in Hela cells (Figure 3). For this purpose we have utilized siRNA to knockdown IKKα, IKKβ and IKKα and IKKβ together in Hela cells. After 3 days of siRNA transfection, knockdown of IKKα and of IKKβ was highly effective. HeLa cells were then treated with TNFα for the indicated times and NF-κB activity was examined through analysis of IκB phosphorylation and degradation. Importantly, kinetics of IκB phosphorylation and degradation in IKKα and IKKβ knock-down cells are both impaired compared to control siRNA treated cells (Figure 3). For instance, 5 min after TNF treatment, there is significant degradation of IκBα in the control cells, while there is little or no loss at that time point in the IKKα or IKKβ knocked-down cells. Additionally, phosphorylation of IκBα is reduced in the IKKα and IKKβ knockdown cells, which is more dramatic given that there are elevated levels of IκBα in these cells at the 5 minute time point. Degradation of IκBα is nearly lost with double-knockdown (Figure 3). To determine the individual roles of IKKα and IKKβ in regulating NF-κB transcriptional activity, knockdown experiments in HeLa cells were combined with transfection of an NF-κB-dependent luciferase reporter (Figure 4). In response to TNF treatment, IKKα and IKKβ each contribute to NF-κB transcriptional activity as measured through reporter assays (Figure 4). These results indicate that IKKα contributes significantly to canonical NF-κB signaling, via control of IκBα phosphorylation and degradation in HeLa cells with subsequent transcriptional stimulation.

### Knockdown of IKKα or IKKβ Diminish TNF-Induced NF-κB Activity in Breast Cancer Cells

**Figure 5 pone-0009428-g005:**
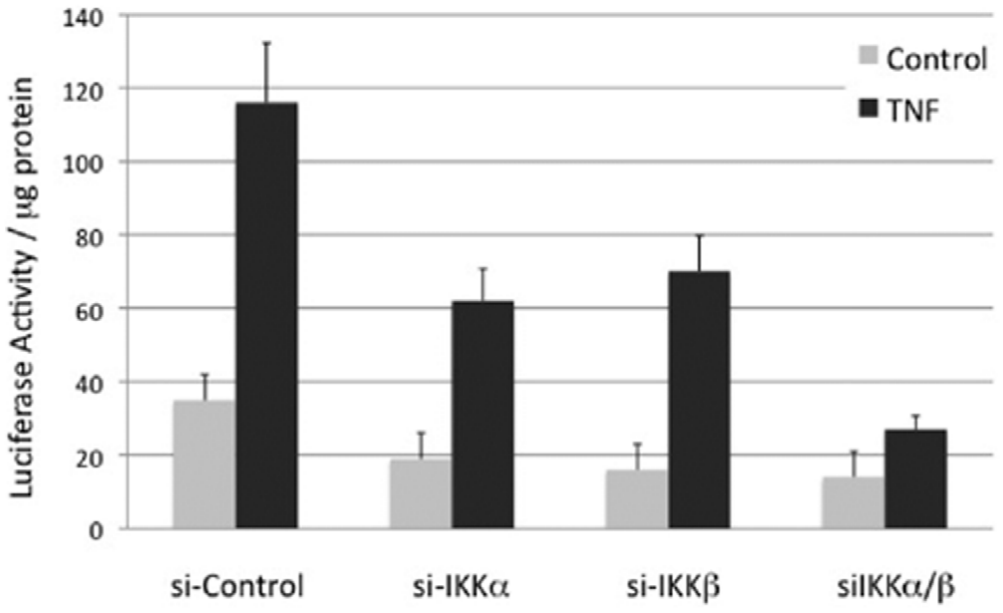
Knockdown of IKKα or IKKβ blocks basal and TNF-α induced NF-κB luciferase activity in breast cancer cells. SKBR3 cells stably expressing 4x-κB firefly luciferase reporter gene were transfected with 100 nM siRNA against IKKα, IKKβ or both. Cells were treated with PBS (black) or 10 ng/ml TNF (gray striped) for 12 hr. Luciferase activity was measured and normalized to total protein levels.

To further analyze the roles of individual IKK kinases on NF-κB activity and to analyze another cell type, we utilized siRNA knockdown of IKKα, IKKβ, and IKKα/β in SKBR3 breast cancer cells (Figure 5). These cells were engineered to stably express an NF-κB-dependent luciferase reporter. siRNA-transfected cells were either left untreated or were treated with TNF. As shown in Figure 5, knockdown of IKKα significantly reduced NF-κB dependent luciferase activity in response to TNF. Comparable reduction was observed with IKKβ knock-down. Importantly, knockdown of IKKα and IKKβ together further reduced the luciferase activity in response to TNF. These results indicate that both IKKα and IKKβ are required for efficient TNF-induced NF-κB activity in breast cancer cells.

### Kinase Inactive IKKβ Inhibits IKKα Activity

**Figure 6:**
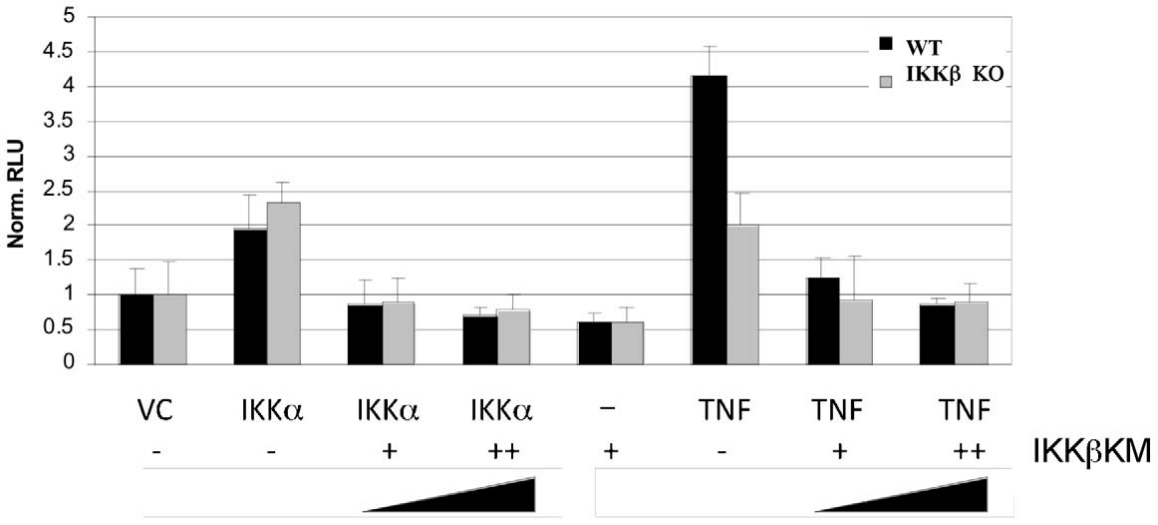
IKKβ kinase mutant inhibits TNF and IKKα-induced NF-κB-dependent reporter gene activity. WT and IKKβ null MEFS were transfected with the indicated vector construct and with the NF-κB-dependent luciferase reporter. Luciferase luciferase activity was measured 48 hr after transfected. Where indicated, cells were treated with TNF for 4 hrs. Relative luciferase values were calculated using a renilla control expression vector for normalization. Relative luciferase values are normalized to vector control samples.

The data presented so far indicate that IKKα as well as IKKβ have significant roles in canonical NF-κB activation. Previous results derived from expression of an IKKβ kinase inactive mutant have suggested that IKKβ activity is highly dominant in canonical NF-κB activation. In order to further examine this hypothesis, we have utilized WT and IKKβ KO MEF cells for transfection studies. WT and IKKβ −/− MEF cells were transfected with empty vector or with an expression vector encoding IKKα along with an NF-κB luciferase reporter plasmid. Results shown in Fig. 6 indicate that IKKα expression activates the NF-κB-dependent reporter in both WT and IKKβ −/− cells, consistent with a role for IKKα in the canonical pathway and demonstrating that IKKα can activate NF-κB in the absence of IKKβ. Co-transfection of the IKKα expression vector with low and higher levels of the IKKβ kinase inactive mutant demonstrates that the kinase inactive form of IKKβ blocks NF-κB-dependent reporter activity in WT and, interestingly, in IKKβ −/− cells. These findings demonstrate that a kinase inactive version of IKKβ inhibits the activity of IKKα. TNF treatment of WT and IKKβ −/− cells showed that cytokine stimulation led to an approximate 4-fold increase in NF-κB-dependent luciferase activity and this response was reduced to approximately 2-fold with the loss of IKKβ (Figure 6). This result is consistent with the findings presented above for reporter activity in cells knocked down for IKKα or IKKβ, and indicate that IKKα plays a key role driving NF-κB activity in the TNF-responsive (canonical) pathway. Expression of the kinase inactive mutant of IKKβ strongly suppressed TNF-induced NF-κB activity in both WT and IKKβ −/− cells. These results further demonstrate that the kinase inactive form of IKKβ suppresses both IKKβ as well as IKKα activity. Therefore studies utilizing IKKβ KM need to be interpreted carefully as the effects observed from IKKβ KM expression will be derived from effects on both IKKβ (as expected) and IKKα (and see discussion). The results from these experiments support the hypothesis that IKKα plays an important role in controlling NF-κB-activity in the canonical pathway.

## Discussion

Based on the phenotypes of IKKα and IKKβ animals, and on results utilizing IKKα −/− and IKKβ −/− MEFs, it has been concluded that IKKβ is the more important IKK catalytic subunit relative to the control of NF-κB activation in the canonical pathway [see 1–5]. Additionally, results using a kinase inactive version of IKKβ have supported these findings since expression of this mutant strongly suppresses NF-κB activation in several cell types. A variety of experiments have implicated NF-κB as a key regulator of human cancer and of diseases associated with inflammation [1]–[5]. Thus, interest in inhibiting NF-κB activation has focused on the development of drugs that block IKKβ. In fact, IKKβ inhibitors have shown efficacy in different models of disease [25], [26]. It is noted that blocking recruitment of IKKγ (NEMO) to the core IKK complex, which blocks canonical IKK activity, has shown broad efficacy in animal models of inflammatory disorders [27]. The experiments presented here indicate that targeting IKKα (alone) or in combination with IKKβ inhibition (via use of distinct IKKα/IKKβ inhibitors, or through blocking IKKγ interaction with the catalytic IKK components) will generate an anti-inflammatory approach. Additionally, inhibiting IKKα alone may have distinct advantages over inhibiting IKKβ since IKKβ inhibition is associated with enhanced release of IL-1 [28].

Original data using knockout MEFs indicated that IKKβ is the critical kinase downstream of TNF in inducing IκBα phosphorylation and degradation [see 13–15, 17–19]. While our data completely agree with those results, loss of IKKα significantly reduced NF-κB activation induced by TNF in MEFs as measured through EMSA (Fig. 2) and reporter assays (Fig. 4–6). Additionally, NF-κB-dependent reporter activity is only partly suppressed in IKKβ−/− cells (Fig. 6), indicating the involvement of IKKα in controlling NF-κB activity in MEFs. The mechanism of IKKα-regulated NF-κB activation is unclear, but may involve the phosphorylation response on p65 where IKKα is clearly involved (see Fig. 1). For the IKKα-controlled pathway, phosphorylation of p65 at ser536 may control DNA binding activity or release from IκB. Interestingly, it was reported that phosphorylation of p65 at ser536 does in fact induce release from IκB without degradation [29]. Additionally, IKKα could potentially induce degradation of IκBβ or IκBε but our analysis did not reveal evidence of this mechanism (data not shown). It has also been reported that IKKα can control IKKβ activity [30], [31], which may contribute to TNF-induced activity in wild-type cells but this cannot explain IKKα activity in IKKβ−/− cells (Fig. 6). Future experiments will address the specific effect whereby IKKα regulates NF-κB activity.

Based on the results obtained in MEFs, we extended our studies to HeLa cells. Using siRNA knockdown of IKKα or IKKβ in these cells, we demonstrate that loss of either IKK subunit suppresses IκBα phosphorylation, and delays IκB degradation (see Fig. 3). These results indicate that in HeLa cells both IKKα and IKKβ are important for IκBα phosphorylation downstream of TNF-induced signaling. The reason that IKKα is not involved in IκBα phosphorylation/degradation in MEFs is unclear at the present time, but is not related to lower relative levels of IKKα in these cells, as determined by immunoblot analysis (see Fig. 1). To analyze another cell type, we utilized SKBR3 human breast cancer cells. Knockdown of IKKα or IKKβ suppressed TNF-induced NF-κB-dependent reporter levels (see Figs. 4 and 5), again supporting the hypothesis that IKKα and IKKβ are both important for TNF-induced NF-κB activation.

We analyzed the effect of expression of a kinase-inactive form of IKKβ on NF-κB-driven reporter gene expression (see Fig. 6). Previously, results derived from utilization of this mutant form of IKKβ have been used to argue the selective involvement of IKKβ in canonical signaling. IKKα expression in WT and in IKKβ −/− cells induces NF-κB reporter activity, which is blocked by expression of IKKβ KM (Fig. 6). The ability of TNF to activate the NF-κB-dependent luciferase reporter is only partly inhibited in IKKβ −/− cells, indicating the involvement of IKKα in the response. Interestingly, expression of the IKKβ kinase mutant strongly suppresses TNF-induced reporter activity (below that seen in IKKβ −/− cells) and blocks TNF-induced in IKKβ −/− cells, indicating that the IKKβ mutant blocks IKKα activity. Thus these results indicate that IKKα is important in the NF-κB-dependent gene expression response to TNF, and that the kinase inactive IKKβ blocks IKKα activity, potentially through engaging a key regulatory molecule upstream of both IKKα and IKKβ or through dimerization with a wild-type IKK subunit and inhibition of the IKK complex.

Why MEFs and HeLa cells appear to utilize IKKα and IKKβ differently regarding effects on IκBα phosphorylation is unclear. This observation may indicate species differences or that different cells/tissues utilize IKKα and IKKβ differently, a concept that should be considered in potential approaches to disease therapy. This latter point may relate to different levels of key upstream regulators of IKK. In this regard, it was reported that knockout of IKKβ in adult hepatocytes did not significantly suppress the ability of TNF to activate NF-κB in these cells, with activity presumably derived from IKKα [22]. This is in contrast to embryonic RelA −/− or IKKβ −/− hepatocytes which are sensitive to TNF-induced killing due to poor activation of NF-κB. Also, this group reported that IKK1/α and IKK2/β cooperate in the canonical pathway in hepatocytes [32]. Furthermore, it was reported that loss of IKKβ leads to a compensatory activation of IKKα [33], but that does not explain why loss of IKKα leads to suppression of NF-κB activity in our studies.

IKKβ inhibitors have been developed and have shown therapeutic responses in different animal models of diseases and are in early clinical trials [25], [26]. These inhibitors show significant preference to IKKβ over IKKα when tested against recombinant proteins. The results presented here indicate that IKKα inhibitors should be developed and tested using animal models of inflammatory diseases. Additionally, the results indicate that dual inhibition of IKKα/β would appear to be an optimal approach to block NF-κB activity downstream of TNF and other inflammatory cytokines [also see 33]. In summary, the data presented here demonstrate that IKKα and IKKβ are both functionally important and cooperate in optimal TNF-induced (canonical) NF-κB activation, with evidence that different cells may utilize IKKα and IKKβ differently.

## References

[1] Gilmore TD (2006). Introduction to NF-kappaB: players, pathways, perspectives.. Oncogene.

[2] Basseres DS, Baldwin AS (2006). Nuclear factor-kappaB and inhibitor of kappaB kinase pathways in oncogenic initiation and progression.. Oncogene.

[3] Hayden MS, Ghosh S (2004). Signaling to NF-kappaB.. Genes Dev.

[4] Ghosh S, Karin M (2002). Missing pieces in the NF-kappaB puzzle.. Cell.

[5] Scheidereit C (2006). IkappaB kinase complexes: gateways to NF-kappaB activation and transcription.. Oncogene.

[6] DiDonato JA, Hayakawa M, Rothwarf DM, Zandi E, Karin M (1997). A cytokine-responsive IkappaB kinase that activates the transcription factor NF-kappaB.. Nature.

[7] Mercurio F, Zhu H, Murray BW, Shevchenko A, Bennett BL (1997). IKK-1 and IKK-2: cytokine-activated IkappaB kinases essential for NF-kappaB activation.. Science.

[8] Zandi E, Rothwarf DM, Delhase M, Hayakawa M, Karin M (1997). The IkappaB kinase complex (IKK) contains two kinase subunits, IKKalpha and IKKbeta, necessary for IkappaB phosphorylation and NF-kappaB activation.. Cell.

[9] Karin M, Ben-Neriah Y (2000). Phosphorylation meets ubiquitination: the control of NF-[kappa]B activity.. Annu Rev Immunol.

[10] Rothwarf DM, Zandi E, Natoli G, Karin M (1998). IKK-gamma is an essential regulatory subunit of the IkappaB kinase complex.. Nature.

[11] Yamaoka S, Courtois G, Bessia C, Whiteside ST, Weil R (1998). Complementation cloning of NEMO, a component of the IkappaB kinase complex essential for NF-kappaB activation.. Cell.

[12] Perkins ND (2006). Post-translational modifications regulating the activity and function of the nuclear factor kappa B pathway.. Oncogene.

[13] Li Q, Van Antwerp D, Mercurio F, Lee KF, Verma IM (1999). Severe liver degeneration in mice lacking the IkappaB kinase 2 gene.. Science.

[14] Li ZW, Chu W, Hu Y, Delhase M, Deerinick T (1999). The IKKβ subunit of IκB kinase (IKK) is essential for NF-κB activation and prevention of apoptosis.. J Exp Med.

[15] Tanaka M, Fuentes ME, Yamaguchi K, Durnin M, Dalrymple S (1999). Embryonic lethality, liver degeneration, and impaired NF-κB activation in IKKβ-deficient mice.. Immunity.

[16] Rudolph D, Yeh WC, Wakeham A, Rudolph B, Nallainathan D (2000). Severe liver degeneration and lack of NF-kappaB activation in NEMO/IKKgamma-deficient mice.. Genes Dev.

[17] Li Q, Lu Q, Hwang JY, Buscher D, Lee KF (1999). IKK1-deficient mice exhibit abnormal development of skin and skeleton.. Genes Dev.

[18] Hu Y, Baud V, Delhase M, Zhang P, Deerinck T (1999). Abnormal morphogenesis but intact IKK activation in mice lacking the IKKalpha subunit of IkappaB kinase.. Science.

[19] Takeda K, Takeuchi O, Tsujimura T, Itami S, Adachi O (1999). Limb and skin abnormalities in mice lacking IKKalpha.. Science.

[20] Yamamoto Y, Verma UN, Prajapati S, Kwak YT, Gaynor RB (2003). Histone H3 phosphorylation by IKK-alpha is critical for cytokine-induced gene expression.. Nature.

[21] Anest V, Hanson JL, Cogswell PC, Steinbrecher KA, Strahl BD (2003). A nucleosomal function for IkappaB kinase-alpha in NF-kappaB-dependent gene expression.. Nature.

[22] Luedde T, Assmus U, Wustefeld T, Meyer zu Vilsendorf A, Roskams T (2005). Deletion of IKK2 in hepatocytes does not sensitize these cells to TNF-induced apoptosis but protects from ischemia/reperfusion injury.. J Clin Invest.

[23] Westerheide SD, Mayo MW, Anest V, Hanson JL, Baldwin AS (2001). The putative oncoprotein Bcl-3 induces cyclin D1 to stimulate G(1) transition.. Mol Cell Biol.

[24] Adli M, Baldwin AS (2006). IKK-i/IKKepsilon controls constitutive, cancer cell-associated NF-kappaB activity via regulation of Ser-536 p65/RelA phosphorylation.. J Biol Chem.

[25] Ziegelbauer K, Gantner F, Lukacs N, Berlin A, Fuchikami K (2005). A selective novel low-molecular weight inhbitor of IKKbeta prevents pulmonary inflammation and shows broads anti-inflammatory activity.. Br J Pharmacol.

[26] Izmailova E, Paz N, Alencar H, Chun M, Schopf L (2007). Use of molecular imaging to quantify response to IKK-2 inhibitor treatment in murine arthritis.. Arthr and Rheum.

[27] Jimi E, Aoki K, Saito H, D'Acquisto F, May M (2004). Selective inhibition of NF-κB blocks osteoclastogenesis and prevents inflammatory bone destruction in vivo.. Nature Med.

[28] Greten FR, Arkan MC, Bollrath J, Hsu LC, Goode J (2007). NF-kappaB is a negative regulator of IL-1beta secretion as revealed by genetic and pharmacological inhibition of IKKbeta.. Cell.

[29] Sasaki C, Barberi T, Ghosh P, Longo D (2005). Phosphorylation of RelA/p65 on Ser536 defines an IκBα-independent activation pathway.. J Biol Chem.

[30] Yamamoto Y, Yin MJ, Gaynor RB (2000). IKKα regulation of IKKβ kinase activity.. Mol Cell Biol.

[31] O'Mahony A, Lin X, Geleziunas R, Greene WC (2000). Activation of the heterodimeric IKKalpha-IKKbeta complex is directional: IKKalpha regulates IKKbeta under both basal and stimulated conditions.. Mol Cell Biol.

[32] Luedde T, Heinrichsdorff J, de Lorenzi R, de Vos R, Roskams T (2008). IKK1 and IKK2 cooperate to maintain bile duct integrity in the liver.. Proc Nat Acad Sci USA.

[33] Lam L, Davis RE, Ngo V, Lenz G, Wright G (2008). Compensatory IKKα activation of classical NF-κB signaling during IKKβ inhibition identified by an RNA interference screen.. Proc Nat Acad Sci U.S.A..

